# Self-selected vs. prescribed aerobic exercise intensity: impacts on pleasure in women with obesity

**DOI:** 10.3389/fpsyg.2025.1533785

**Published:** 2025-02-07

**Authors:** Carlos Eduardo Rosa Da Silva, Wilian Jesus Santana, Gustavo Almeida, Adriano Verame, Antonio Roberto Doro, Eduardo Barbosa, Leonardo Lima, Helton Magalhães Dias, Marcelo Callegari Zanetti, Aylton Figueira Junior

**Affiliations:** GETAFIS - São Judas Tadeu University, São Paulo, Brazil

**Keywords:** psychophysiological responses, enjoyment, motivation, physical activity, small group training

## Abstract

**Introduction:**

Self-selection of aerobic training intensity is an approach that allows practitioners to develop autonomy (choice of pace), improve physical fitness, and contribute to better affective responses and training adherence. However, it is still unclear whether self-selection of training intensity in group settings is comparable to individual training conditions. The aim of the present study was to compare the effect of three aerobic training protocols on psychophysiological responses in physically inactive adult obese women.

**Methods:**

The sample consisted of 90 women with a mean age of 48.3 ± 5.5 years and BMI of 31.2 ± 4.8 kg/m^2^, who participated in three treadmill aerobic training protocols: 1- Control group (IPI) with individually prescribed intensity (64 to 76% of HRmax), 2- Small group (SGS) with self-selected intensity, and 3- Individual with self-selected intensity (ISS). Heart rate (HR), total session volume (VL), affective valence was determined by feeling scale (FS), enjoyment was determined by Physical Activity Enjoyment Scale (PACES), intention to repeat the exercise session (INT), and Basic Psychological Needs Satisfaction Scale (BPNFS) were evaluated. Statistical analyses were conducted using two-way ANOVA for HR and FS, and one-way ANOVA for VL, PACES, INT, and BPNFS with Tukey *post hoc* test and significance criteria (*p* < 0.05).

**Results:**

The results showed that HR and VL were similar (*p* > 0.05) between the protocols IPI, SGS and ISS, while FS, PACES, and INT were significantly higher in SGS and ISS than IPI protocols (*p* < 0.05). BPNFS also presented better results for the SGS and ISS protocols (*p* < 0.05).

**Discussion:**

These findings suggest that the intensity and training volume were similar among different protocols. Affective responses were more positive perceived in SGS and ISS protocols.

**Conclusion:**

We conclude that intensity self-selection is a safe, effective, and comparable strategy to prescribed intensity at moderate levels, providing a more enjoyable experience that may contribute to greater adherence to aerobic training.

## 1 Introduction

Aerobic training has been recommended to reduce overweight and obesity as risk factors for, metabolic and cardiovascular diseases, as well some types of cancer (Shilton et al., 2024). Overweight and obesity subjects have a 6-fold higher risk of developing type 2 diabetes and 3-fold higher risk of developing cardiovascular diseases compared to normal weight adults (Zhang et al., 2024). On the other hand, healthy lifestyle based on regular physical activity/exercise reduce overweight and obesity associated risks over 25 chronic diseases(Zhang et al., 2024; Khan et al., 2018; Hruby and Hu, 2015; Blüher, 2019), contribute to healthier aging (Shilton et al., 2024; Stafie et al., 2024; Albini et al., 2025; Elagizi et al., 2020; Yang and Petrini, 2018).

Physical inactivity contributes to the development of overweight and obesity, and negatively impact on health and quality of life (Almeida et al., 2024; Sacramento et al., 2024; Guthold et al., 2020). Globally, 7.2% of cardiovascular disease and 7.6% all-cause mortality are attributable to physical inactivity (Katzmarzyk et al., 2022). Psychophysiological determinants, such as motivation and physical limitations, are significant barriers to physical activity practice (Azizan and Fadzil, 2024).

Adherence to exercise programs in fitness centers has shown drop out between 40 and 70% of new participants within 3–6 months after starting (Sperandei et al., 2016; Gjestvang et al., 2019; Gjestvang et al., 2020; Gjestvang et al., 2021; Gjestvang et al., 2023). It suggests that current strategies to engagement have not been effective in addressing behavioral demands, as lack of intrinsic motivation, absence of personalized support, and enjoyment perception in training sessions (Sperandei et al., 2016; Gjestvang et al., 2019; Gjestvang et al., 2020; Gjestvang et al., 2021; Gjestvang et al., 2023; Clavel San Emeterio et al., 2019; Faro et al., 2023; Rodrigues et al., 2021).

Traditional exercise prescription models, as outlined in the guidelines (Liguori et al., 2021), prioritize safety and effectiveness but often fail to ensure long-term adherence. This has led to the proposal of a tripartite model that incorporates more enjoyable exercise doses to enhance engagement and promote greater future participation in training sessions (Ladwig et al., 2017; Ekkekakis and Tiller, 2022; Ekkekakis, 2017; Teixeira et al., 2012). Emotional factors, such as enjoyment during and after exercise and the satisfaction of achieving goals, are crucial motivators (Wienke and Jekauc, 2016), highlighting the need for strategies that foster positive emotional experiences (Rodrigues et al., 2021; Liguori et al., 2021; Ladwig et al., 2017; Teixeira et al., 2012; Wienke and Jekauc, 2016; Rodrigues et al., 2021; Teixeira et al., 2022; Ekkekakis et al., 2011; Williams et al., 2016). The Dual-Mode Theory (Ekkekakis, 2009) explains affective responses during aerobic exercise through two systems: automatic perception and cognitive control. As intensity increases beyond the ventilatory threshold, pleasure generally decreases. Positive affective responses can improve adherence, while negative experiences, such as discomfort, may lead to dropout (Williams, 2008; Ekkekakis and Lind, 2005; Parfitt et al., 2006; Hall et al., 2005).

Affective responses, whether pleasurable or discomforting, are central to the motivation for physical exercise. Behaviors with a greater perception of pleasure are more likely to be repeated, while displeasure may discourage engagement. Aerobic exercise intensities that generate positive emotions can contribute to greater adherence, whereas excessive effort or displeasure may lead to dropout. Therefore, exercise prescriptions should consider not only physiological benefits but also emotional responses, as these influence the decision to continue or discontinue training (Ladwig et al., 2017; Ekkekakis, 2003; Ekkekakis and Petruzzello, 1999; Rhodes and Kates, 2015; Williams and Evans, 2014). Affective valence, the spectrum of feelings ranging from pleasure to displeasure during activity, spans sensations that can enhance participation to those that discourage engagement (Russell, 2003). Emotions influence behavior through feedback, automaticity, and reflection; understanding these mechanisms is essential to comprehending how emotions can impact long-term adherence to physical exercise (Baumeister et al., 2007).

Positive social interactions and a supportive environment are crucial for promoting adherence to training programs (Gjestvang et al., 2020; Gjestvang et al., 2021; Gjestvang et al., 2023; Gabay and Oravitan, 2022; Withall et al., 2022). Small group exercise, a trend in fitness (Martinez Kercher et al., 2022), can enhance socialization and motivation, fostering a sense of community, as explained by Self-Determination Theory (Martinez Kercher et al., 2022; Wayment and McDonald, 2017; Spink et al., 2010; Golaszewski et al., 2022; Deci and Ryan, 2000). This theory identifies three psychological needs—autonomy, competence, and relatedness—as key elements of motivation. Autonomy, supported by self-selected exercise intensity, increases the sense of control and can improve affective responses, such as the perception of pleasure (Ekkekakis and Lind, 2005; Parfitt et al., 2006; Deci and Ryan, 2000; Ntoumanis et al., 2021). Together, these factors may influence the long-term maintenance of physical exercise practice, as individuals are more likely to continue activities aligned with their preferences and abilities (Williams and Evans, 2014; Ekkekakis et al., 2008; Lind et al., 2008).

Self-selected intensity promotes greater autonomy, allowing individuals to adjust their effort to align with their preferences. This approach is generally associated with more positive affective responses, such as enhanced pleasure and well-being (Parfitt et al., 2006). Self-selected intensity in aerobic training is effective in improving physiological and psychological aspects in different populations, such as obese women, adolescents and physically inactive older adults (Yang and Petrini, 2018; Barros et al., 2019; Alves et al., 2022). Physiological and psychological responses, including the perception of pleasure, in women with overweight or obesity during physical exercise is crucial for understanding the factors that influence adherence and motivation within this population.

Although previous studies (Ekkekakis, 2009; Ekkekakis and Lind, 2005; Lind et al., 2008; Ekkekakis et al., 2006) have investigated the differences between self-selected and prescribed exercise intensities, no prior research has compared self-selected protocols in small groups with individually prescribed ones, highlighting a significant gap in the literature.

The purpose of the present study is to compare the psychophysiological responses of overweight and obese women during aerobic training prescribed using two different methods: (A) prescribed intensities and (B) self-selected intensities, both in individual and small group settings. The primary hypothesis is that the intensity and training volume in prescribed group protocols will be similar to those in self-selected group protocols, both in individual and small group contexts. The secondary hypothesis is that self-selected intensity protocols will elicit more positive affective responses, such as higher affective valence, greater enjoyment, and increased intention to repeat exercise sessions, consistent with hedonic theory (Ekkekakis, 2003; Kahneman, 1999), which suggests that more pleasurable behaviors are more likely to be repeated. Additionally, it is expected that self-selected intensity groups will promote a greater perception of autonomy compared to the prescribed intensity group, as the possibility of choice is a central component of satisfying the need for autonomy, according to Self-Determination Theory (Deci and Ryan, 2000). Small group training is anticipated to promote a greater perception of relatedness compared to other groups, addressing the need for social connection and mutual support, while the perception of competence is expected to be similar across groups, as it relates to participants’ ability to accomplish the proposed tasks, aligning with the psychological need to feel effective and capable in their efforts (Deci and Ryan, 2000). These connections to basic psychological needs may explain how different protocols influence motivation and contribute to more positive experiences in aerobic exercise practice (Rodrigues et al., 2021; Ekkekakis, 2003; Rhodes and Kates, 2015; Ekkekakis et al., 2005; Lind et al., 2005).

## 2 Methods

### 2.1 Study design

A single-blind, randomized controlled trial was conducted, involving three experimental groups. All training sessions were standardized and conducted between 8:00 AM and 12:00 PM under identical conditions, overseen by the same evaluator, and performed in a temperature-controlled environment set at 23°C.

### 2.2 Subjects

Eligible participants were women aged 40 to 65 years who were apparently healthy, had no contraindications to physical exercise, and had not engaged in structured physical activity or sports within the past 12 months. Exclusion criteria included high-risk cardiovascular disease stratification, as defined by the American College of Sports Medicine (Liguori et al., 2021), and/or hypertension (blood pressure > 130/80 mmHg) measured prior to the intervention. The selection of women as the population in the present study is justified by their greater vulnerability to physical inactivity, and health risks related to overweight and obesity, which requires specific interventions (Guthold et al., 2018; Strain et al., 2024; Mayo et al., 2019; Mielke et al., 2018). Participants were recruited via social media platforms (Instagram and Facebook) through informational videos highlighting the health benefits of exercise. Eligibility criteria included physical activity levels below 150 min per week (Matsudo et al., 2001) and a body mass index (BMI) greater than 25 kg/m^2^.

### 2.3 Interventions

#### 2.3.1 Pre-exercise evaluation and exercise program

Data collection was conducted over multiple sessions. On the first session, body mass (kg) and height (cm) were measured using a Sanny BL201PP digital scale with an integrated stadiometer, and resting blood pressure was assessed using an OMROM 7122 arm blood pressure monitor. Participants were familiarized with the treadmill (MOVEMENT RT-250 model) and psychometric instruments, and completed a physical activity risk screening questionnaire (Thomas et al., 1992), and provided all necessary medical authorizations.

The study was conducted using a treadmill to provide a more rigorous control of variables such as intensity and volume, ensuring consistency across sessions. Additionally, the treadmill allowed for standardized conditions that facilitated the collection of psychometric scales during the protocol, minimizing external influences and enhancing the reliability of the measurements. This controlled environment ensured precise monitoring and evaluation of the participants’ responses throughout the exercise session.

**Figure 1:**
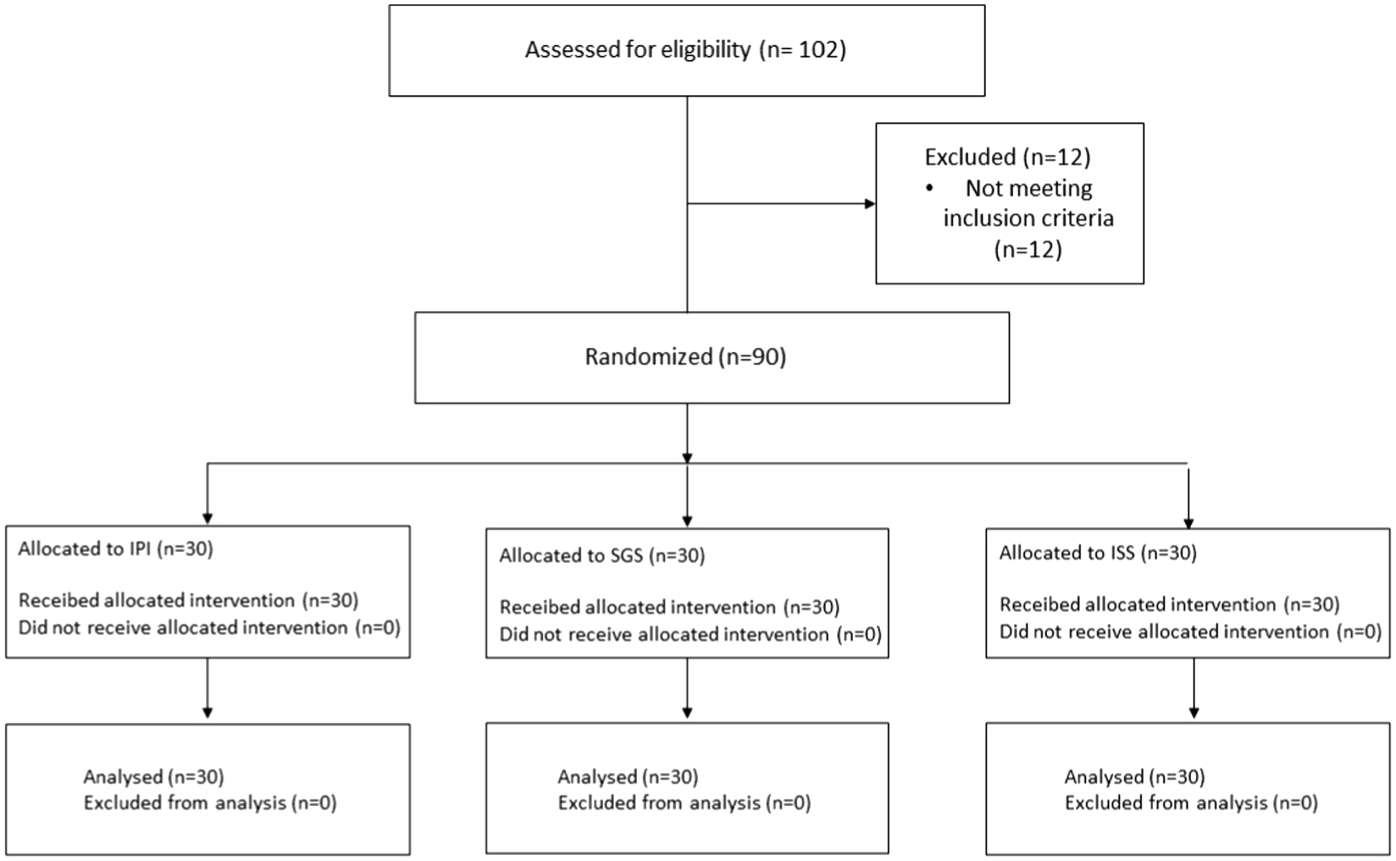
CONSORT flow diagram.

During the second session, an incremental treadmill test was performed to determine maximum heart rate and to calculate training zone percentages (Ellestad et al., 1969). Participants were then randomized and allocated into three experimental groups. The third session was dedicated to the implementation of the aerobic training protocols, as outlined in Figure 1.

Upon arrival at the laboratory, each participant remained seated for 10 min in silence, avoiding verbal or visual interaction with others, and refrained from using mobile phones or electronic devices prior to the test. The psychometric scales and questionnaires were thoroughly explained and reviewed to ensure the reliability and reproducibility of the measurements.

This study evaluated three distinct aerobic training protocols: (Shilton et al., 2024) Individual with Prescribed Intensity (IPI), corresponding to 64–76% HRmax as recommended by the American College of Sports Medicine (ACSM) for improving cardiorespiratory fitness in sedentary individuals (Liguori et al., 2021; Zhang et al., 2024) Individual with Self-Selected Intensity (ISS); and (Khan et al., 2018) Small Group with Self-Selected Intensity (SGS).

In the IPI protocol, participants performed 30 min of aerobic exercise on a treadmill at a prescribed intensity of 64–76% HRmax. This intensity range is classified as moderate and sufficient for enhancing cardiorespiratory fitness, according to ACSM guidelines (Liguori et al., 2021). The training was conducted in a controlled environment, with the participant and the researcher only.

In the ISS protocol, participants performed 30 min of aerobic exercise on a treadmill at an intensity they self-selected. The treadmill speed was determined autonomously by each participant, who adjusted the pace as they deemed appropriate to complete the session. Participants could increase or decrease the speed at any point during the training, and the session was conducted in the presence of the participant and the researcher only.

The SGS protocol involved three participants performing 30 min of aerobic exercise simultaneously on individual treadmills. Each participant chose their training intensity, maintaining a pace they considered comfortable. The treadmills were positioned to prevent participants from viewing each other’s control panels, thereby minimizing external influence on individual intensity selection.

For the ISS and SGS protocols, participants were instructed using the standardized phrase: *“Choose the pace you believe is appropriate to complete 30 min of physical exercise on the treadmill.”* In both protocols, participants were explicitly instructed to do not verbalize their ratings on psychometric scales to prevent influencing one another. Instead, they pointed to and selected values independently.

The ISS and SGS protocols were designed to enhance the participants’ affective experiences by allowing self-selected intensities. In the SGS group, participants were encouraged to engage in conversation during the training about any topic, fostering social interaction as a strategy to improve the affective experience during exercise (Teixeira et al., 2012; Ntoumanis et al., 2021). Conversations included perceptions and feelings about the exercise session and individual pace choices.

The IPI adhered to standard guidelines as prescribed by the ACSM (Liguori et al., 2021). Participants in all groups were blinded to the specific objectives of the study, particularly regarding the potential impact of the instructions on their affective experiences. The description of parameters such as intensity, environment and participants are shown in Table 1.

**Table 1:** Description of the characteristics of each protocol.

	*N*	Intensity	Duration	Environment	Participants
IPI	30	64–76% HRmáx	30 min	Individual	1
SGS	30	Self-selected	30 min	Small group	3
ISS	30	Self-selected	30 min	Individual	1

IPI, Group with prescribed intensity (64–76% HRmax) (Liguori et al., 2021); SGS, Small Group with self-selected intensity; ISS, Individual Group with self-selected intensity. N, Number of participants in each group.

The differences in protocol lie in the need to evaluate how self-selected intensity, could promote greater autonomy and pleasure, and impact on psychophysiological and affective responses compared to prescribed intensity, especially in populations more vulnerable to physical inactivity, such as women with obesity.

### 2.4 Randomization

Given the continuous recruitment design of the study, a simple randomization procedure was employed. Participants were blinded to their group allocation and the specific characteristics of the interventions being compared. Randomization was carried out by researchers CERS and AFJ, who were the only team members directly responsible for implementing the exercise interventions and, consequently, aware of each participant’s group assignment. The researchers responsible for data screening and statistical analysis had no contact with the participants and remained blinded to group allocations throughout the study. Participants were randomized into three groups: IPI – Prescribed Intensity (64–76% HRmax, n = 30), Individual with Self-Selected Intensity (ISS, n = 30), and Small Group with Self-Selected Intensity (SGS, n = 30). The process of randomization was carried out randomly, ensuring unbiased allocation of participants across the three groups. Given the homogeneity of the sample characteristics, such as BMI and age, the randomization process resulted in comparable baseline attributes among the groups. This methodological approach minimized potential confounding factors, ensuring that the observed differences in outcomes could be attributed to the intervention protocols rather than pre-existing disparities between the groups.

### 2.5 Evaluation time points of the exercise session

The variables of interest were assessed before, during, and after each exercise session across all groups.

In all protocols, heart rate (HR) was continuously monitored throughout the training session. The Feeling Scale (FS) (Guthold et al., 2018) was administered five times: at rest, at the 5th, 15th, and 25th minutes of the session, and 15 min post-training. Following the completion of each protocol, participants remained seated for 15 min during the recovery period. During this time, the FS, the Physical Activity Enjoyment Scale (PACES) (Strain et al., 2024), and the Intention to Repeat the Exercise Session Scale (INT) (Mayo et al., 2019) were applied, in accordance with established guidelines for aerobic exercise analysis (Mielke et al., 2018).

Psychometric assessments were conducted without verbal expression of perceptions to minimize interference with interpersonal decision-making processes, such as vicarious experience, fear of external judgment, or anxiety.

Volume load (VL) was recorded at the end of each protocol.

**Figure 2:**
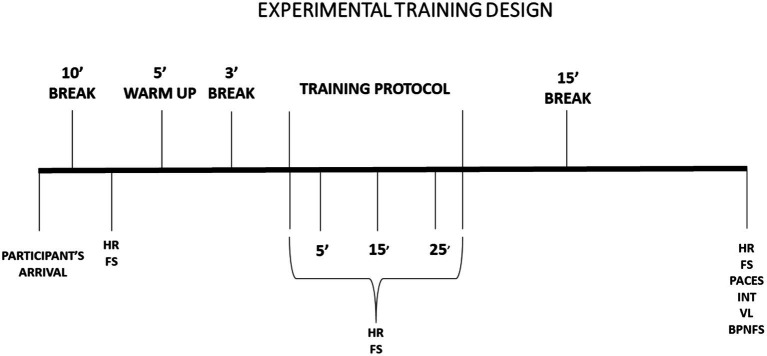
Experimental design of the study in different aerobic training protocols. HR, Heart rate (bpm); FS, Feeling Scale; PACES, Physical Activity Enjoyment Scale; INT, Intention to Repeat the Exercise Session Scale; VL, total session volume; BPNSFS, Basic Psychological Needs Satisfaction and Frustration Scale.

Data collection followed the experimental procedures outlined in Figure 2.

Affective responses during exercise may vary, in obese individuals potentially experiencing more positive feelings when the exercise is adapted to their preferences and abilities (Freitas et al., 2015).

### 2.6 Instruments

#### 2.6.1 Heart rate

Heart rate was monitored continuously throughout the entire protocol using the POLAR H7 heart rate monitor, connected via Bluetooth to the POLAR TEAM app. Relative intensity was calculated based on each participant’s maximum heart rate (HRmax), determined through an incremental treadmill test (Ellestad et al., 1969). HR measurements were recorded at five time points: at rest, at the 5th, 15th, and 25th minutes during the protocol, and 15 min post-protocol.

#### 2.6.2 Feeling scale

The affective valence response to exercise was assessed using the Feeling Scale (Hardy and Rejeski, 1989), an 11-point bipolar scale ranging from +5 (“very good”) to −5 (“very bad”), reflecting pleasure and displeasure, respectively. At the beginning of each protocol, participants were instructed: *“Some individuals experience pleasure during exercise, while others experience displeasure. This perception is individual. How do you evaluate your perception of pleasure or displeasure at this moment in the exercise?”* Measurements were taken at five time points: at rest, at the 5th, 15th, and 25th minutes during the protocol, and 15 min post-protocol, resulting in a total of five measurements per protocol (Ekkekakis et al., 2023). The FS is closely associated with Hedonic Theory (Ekkekakis, 2003; Ekkekakis et al., 2006), which suggests that individuals are more likely to engage in behaviors that enhance pleasure and reduce discomfort. In the context of exercise, the affective valence assessed by the FS facilitates the identification of intensities and conditions that promote positive affective experiences. This information is particularly valuable for designing individualized exercise prescriptions, especially for vulnerable populations, such as obese and physically inactive women, who may exhibit heightened sensitivity to discomfort during physical activity.

#### 2.6.3 Physical activity enjoyment scale

The Physical Activity Enjoyment Scale is an 18-item instrument designed to assess enjoyment during exercise or physical activity (Kendzierski and DeCarlo, 1991). It includes 12 negatively worded items and 6 positively worded items, with responses rated on a 1–7 bipolar scale. Participants were instructed: *“How do you feel right now regarding the exercise or physical activity?”* Data collection occurred at the conclusion of each protocol. This instrument is based on the Hedonic Theory (Ekkekakis, 2003; Ekkekakis et al., 2006), which suggests that individuals are more likely to repeat behaviors that maximize pleasure and minimize discomfort, thus enabling the identification of practices that foster positive experiences.

#### 2.6.4 Intention to repeat the exercise session scale

Participants’ intention to engage in future exercise during the following week was assessed using a 2-item scale, adapted from previous studies (Jung et al., 2014; Kwan and Bryan, 2010) and translated into Portuguese (Gomes and Capelão, 2013). The scale included the question: *“I intend to perform this exercise I did today at least three times next week.”* Responses were recorded on a 7-point Likert scale, with anchors ranging from 1–*“very unlikely”* to 7–*“very likely.”* The scale was administered at the conclusion of each protocol. The hedonic theory (Ekkekakis, 2003; Kahneman, 1999) suggests that more pleasurable behaviors are more likely to be repeated. The use of the scale allows for assessing how positive or negative experiences during the exercise session influence the intention to continue.

#### 2.6.5 Basic psychological needs satisfaction and frustration scale

Basic psychological needs—Autonomy, Relatedness, and Competence—were assessed using the Basic Psychological Needs Satisfaction and Frustration Scale (Murphy et al., 2022). The scale consists of 24 items, equally distributed among the three constructs and their satisfaction and frustration dimensions. Each item is rated on a Likert scale from 1 (strongly disagree) to 5 (strongly agree), yielding scores ranging from 6 to 30 for each need, based on the number of items. This instrument enables the investigation of how the fulfillment of basic psychological needs, as outlined in Self-Determination Theory (Deci and Ryan, 2000), may mediate affective responses during exercise, thereby enhancing pleasure and promoting long-term adherence to training programs.

#### 2.6.6 Volume load

At the end of each protocol, the total distance covered was recorded to characterize the volume load of the training session.

The present study did not assess training adherence, as it employed a cross-sectional design that examined psychophysiological responses during a single acute aerobic training session. While the study offers insights into psychological variables, it merely hypothesizes their potential association with long-term adherence. Further longitudinal studies are required to establish how these variables influence sustained engagement in exercise programs.

### 2.7 Statistical analysis

Sample size was calculated *a priori* using G*Power (v.3.1.9.7) (Faul et al., 2009). A split-plot ANOVA (2 groups × 5 time points) was performed. Assuming an effect size of *f* = 0.25 for the interaction, with *α* = 0.05, statistical power of 1 – *β* = 0.95, a correlation between repeated measurements of r = 0.50, and a violation of sphericity (*ε* = 1), a total sample size of 39 participants (13 per group) was required. Changes in percentage (*Δ*%) were used to assess differences between group means.

After initial data screening, descriptive analyses (means and standard deviations) and Levene’s test for data normality were performed.

For subsequent analyses, SPSS 25.0 (SPSS, Inc., Chicago, IL) and GraphPad Prism 8.0.2 (263) were used for graph construction.

HR and FS variables were analyzed using a two-way ANOVA (3 groups × 5 time points). The PACES, INT, and BPNSFS variables were evaluated using one-way ANOVA. When violations of sphericity were detected (i.e., for analyses involving more than two time points for the within-subjects factor), Greenhouse–Geisser corrections were applied to the degrees of freedom. For significant main effects and interactions, the Tukey *post hoc* test was performed with a significance level set at *p* < 0.05. Eta-squared (η^2^) effect sizes were calculated and interpreted following Cohen’s guidelines (Cohen, 1988), with “small” (0.01), “medium” (0.06), and “large” (0.14) effect size thresholds. Mean differences and their corresponding 95% confidence intervals (CI) are presented.

### 2.8 Ethics statement

The studies involving humans were approved by CNS Resolution No. 466/12, in accordance with ethical principles standardized by São Judas University Ethics Committee N°: 4.583.831. The studies were conducted in accordance with the local legislation and institutional requirements. The participants provided their written informed consent to participate in this study. Written informed consent was obtained from the individual(s) for the publication of any potentially identifiable images or data included in this article.

## 3 Results

A total of 102 participants were assessed for eligibility. Twelve participants were excluded for failing to meet the inclusion criteria (BMI >25 kg/m^2^, n = 12; age > 40 years). Ninety participants were randomly assigned to the following groups: Control (IPI, n = 30), Individual with Self-Selected Intensity (ISS, n = 30), and Small Group with Self-Selected Intensity (SGS, n = 30). The recruitment process and protocol implementation took place over a period of 18 months. All data were collected prior to the end of June 2024.

The descriptive characteristics of the sample are presented in Table 2. The groups exhibited only minor differences in age and BMI. The SGS group had a lower mean age and a higher standard deviation compared to the other groups.

**Table 2:** Descriptive statistics of sample characteristics.

	Total sample (*n* = 90)		IPI (*n* = 30)		SGS (*n* = 30)		ISS (*n* = 30)	
	M	SD	M	SD	M	SD	M	SD
Age	48.30	5.50	48.00	5.40	45.50	8.80	49.20	6.00
BMI (kg/m2)	31.20	4.80	30.00	4.90	31.30	4.30	32.20	5.20
VO2 peak	25.90	3.10	26.20	3.10	26.60	4.00	24.80	1.10

n, sample size; *M*, mean; SD, standard deviation.

Heart rate data are presented in Table 3, with the mean HR during the exercise session remaining within the moderate intensity range for all groups: IPI = 70.5% HRmax, SGS = 70.3% HRmax, and ISS = 71% HRmax. The two-way ANOVA revealed a minimal interaction effect between group and time across the different groups (*F* = 1.17, *p* = 0.31, η^2^ = 0.02).

**Table 3:** Descriptive statistics, group differences, and effect sizes for heart rate (bpm) of each protocol.

	**IPI (*n* = 30)**			**SGS (*n* = 30)**				**ISS (*n* = 30)**				**Group by time interaction**		
	***M***	**SD**	**(% Max HR)**	***M***	**SD**	**(% Max HR)**	**Δ%**	***M***	**SD**	**(% Max HR)**	**Δ%**	***F* value**	***p* value**	**η** ^**2**^
HR before	77.83	12.83	45.3%	80.60	12.33	46.9%	0.04	84.27	9.98	49.0%	0.08	1.17	0.31	0.02
HR 5 min	117.87	18.70	68.5%	112.70	12.67	65.5%	−0.04	118.30	8.01	68.8%	0.00			
HR 15 min	122.60	12.88	71.3%	125.97	8.3	73.2%	0.03	125.20	14.41	72.8%	0.02			
HR 25 min	123.30	12.72	71.7%	124.00	14.11	72.1%	0.01	122.93	18.21	71.5%	0			
HR session	121.26	3.41	70.5%	120.89	3.12	70.3%	0	122.14	5.15	71.0%	0.01			
HR after	89.33	14.69	51.9%	88.37	14.18	51.4%	−0.01	86.50	14.00	50.3%	−0.03			

**Mean difference (95% CI)**				**Mean difference (95% CI)**			
MD	LBCI	UBCI	*p*-value	MD	LBCI	UBCI	*p*-value
−0.21	−3.81	3.38	0.98	1.32	−4.92	2.27	0.66

IPI, Group with prescribed intensity (64–76% HRmax) (Liguori et al., 2021); SGS, Small Group with self-selected intensity; ISS, Individual Group with self-selected intensity. M, mean; SD, standard deviation; Δ%, % difference in M compared to the control group, *F* value, *F*-ratio value; *p* value, probability of *F*; η^2^, eta-squared effect size; CI, Confidence interval; LBCI, Lower bound of the 95% confidence interval; UBCI, Upper bound of the 95% confidence interval; MD, mean difference; HR, heart rate (bpm); HR Session, HR during the session, without considering before and after exercise.

**Figure 3:**
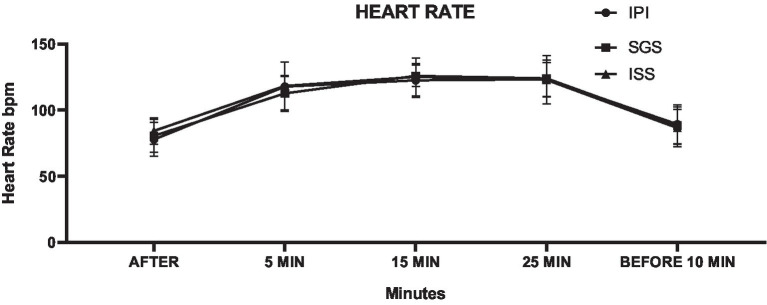
Means and standard deviations of heart rate (HR) (vertical axis) compared between the control and experimental groups and collection time points (horizontal axis).

The mean differences also did not reveal significant variations between the ISS and SGS groups when compared to the IPI group. The SGS group exhibited an average HR that was 0.3% lower than the IPI group (*p* = 0.98, mean difference = −0.21, 95% CI -3.81 to 3.38), while the ISS group had an average HR that was 0.7% higher than the IPI group (*p* = 0.66, mean difference = 1.32, 95% CI -4.92 to 2.27). These results support the primary hypothesis. The findings suggest that self-selected intensity is comparable to prescribed intensity, both classified as moderate (Liguori et al., 2021), and represent a safe and effective training approach for adult women beginning aerobic exercise programs, particularly those who are overweight or obese, as illustrated in Figure 3.

The volume load analyses of the aerobic training session are presented in Table 4. The one-way ANOVA showed a small interaction effect among the different groups (*F* = 0.87, *p* = 0.45, η^2^ = 0.03).

**Table 4:** Descriptive statistics, group differences, and effect sizes for training volume (km) of each protocol.

				Mean difference (95% CI)						
	*M*	SD	Δ%	MD	LBCI	UBCI	*p*-value	F-value	*p*-value	η^2^
**IPI**	3.14	0.30						0.87	0.45	0.03
**SGS**	3.02	0.27	−3.8%	0.08	−0.72	0.23	0.28			
**ISS**	3.05	0.32	−2.9%	0.12	−0.35	0.27	0.12			

IPI, Group with prescribed intensity (64–76% HRmax) (Liguori et al., 2021); SGS, Small Group with self-selected intensity; ISS, Individual Group with self-selected intensity. M, mean; SD, standard deviation; Δ%, % difference in M compared to the control group; *F* value, *F*-ratio value; *p*-value, probability of F; η^2^, eta-squared effect size; CI, confidence interval; LBCI, Lower bound of the 95% confidence interval; UBCI, upper bound of the 95% confidence interval; MD, mean difference; HR, heart rate; HR Session, HR during the session, without considering before and after exercise.

**Figure 4:**
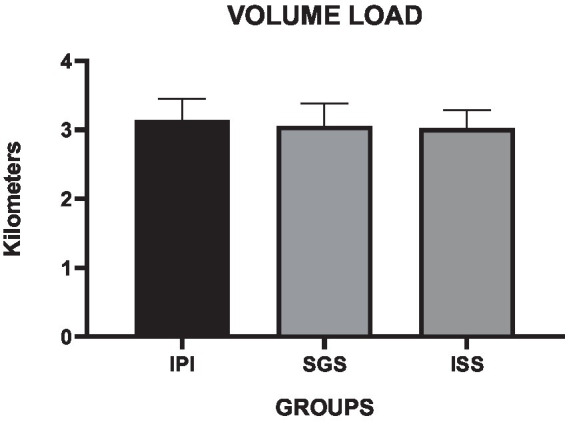
Means and standard deviations of the volume load of the exercise session (vertical axis) compared between the control and experimental groups (horizontal axis).

The mean values also demonstrated similarities between the groups. The SGS group exhibited a 3.8% lower mean volume load recorded at the end of the exercise session (*p* = 0.28, mean difference = 0.08, 95% CI = −0.72 to 0.23), while the ISS group had a mean VL that was 2.9% lower compared to the IPI group (*p* = 0.12, mean difference = 0.12, 95% CI = −0.35 to 0.27). In support of our secondary hypothesis, the VL results were similar across the groups. These findings suggest that self-selected intensity is comparable to prescribed intensity regarding the total distance covered during the exercise session, as illustrated in Figure 4.

The analyses of the mean values of the Feeling Scale during the exercise session are presented in Table 5. Significant differences were found between the SGS and ISS compared to the IPI protocol, showing a moderate interaction effect between group and time across the different groups (*F* = 5.72, *p* = 0, η^2^ = 0.09).

**Table 5:** Descriptive statistics, group differences, and effect sizes for the Feeling Scale (11 points) of each protocol.

	IPI (*n* = 30)		SGS (*n* = 30)			ISS (*n* = 30)			Group by time interaction		
	*M*	SD	*M*	SD	Δ%	M	SD	Δ%	F value	*p*-value	η^2^
FS before	4.70	0.60	4.63	0.76	−1.4%	4.50	0.86	−4.3%	5.72	0	0.09
FS—5 min	2.23	1.94	3.70	1.06	65.7%	4.20	1.00	88.1%			
FS—15 min	1.50	1.57	2.60	1.43	73.3%	3.53	1.28	135.6%			
FS—25 min	1.60	1.48	2.93	1.68	83.3%	2.87	1.11	79.2%			
FS session	1.78	0.25	3.08	0.31	73.1%	3.53	0.14	98.8%			
FS after 10 min	4.33	0.92	4.80	0.41	10.8%	4.60	0.81	6.2%			

**Mean difference (95% CI)**			**Mean difference (95% CI)**		
MD	LBCI	UBCI	MD	LBCI	UBCI
−0.78	−1.1	−0.45	−1.02	−1.34	−0.7

IPI, Group with prescribed intensity (64–76% HRmax) (Liguori et al., 2021); SGS, Small Group with self-selected intensity; ISS, Individual Group with self-selected intensity. M, mean; SD, standard deviation, Δ%, % difference in M compared to the control group; F, F-ratio value; p, probability of F; η^2^, eta-squared effect size; CI, confidence interval; LBCI, lower bound of the 95% confidence interval; UBCI, upper bound of the 95% confidence interval; MD, mean difference; FS Session, FS during the session, without considering before and after exercise.

**Figure 5:**
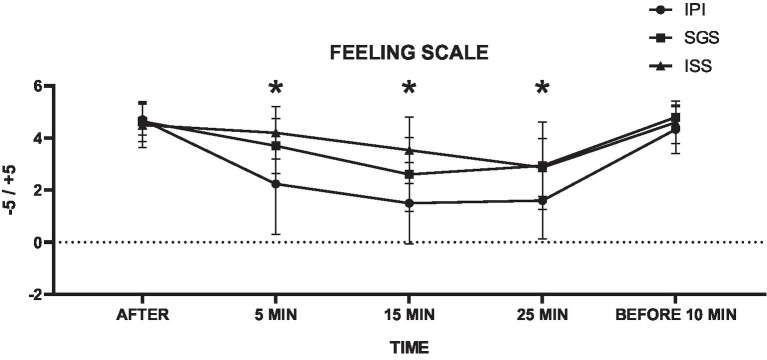
Means and standard deviations of the Feeling Scale (FS) (vertical axis) compared between the control and experimental groups and collection time points (horizontal axis). * = difference *p* < 0.05 compared to the IPI group.

The SGS group demonstrated a 73.1% increase in mean Feeling Scale scores, excluding pre- and post-exercise session values (*p* = 0, mean difference = −1.1, 95% CI = −1.1 to −0.45), while the ISS group showed a 98.8% higher FS compared to the IPI group (p = 0, mean difference = −1.02, 95% CI = −1.34 to −0.7). In support of our secondary hypothesis, the FS results were significantly higher in both the SGS and ISS groups. These findings suggest that self-selected intensity is more enjoyable in both individual and small group settings. This indicates a more positive affective experience for women beginning physical training programs, particularly those who are overweight or obese, as shown in Figure 5.

A moderate effect was observed on the Physical Activity Enjoyment Scale, as presented in Table 6, indicating a significant interaction among the different groups (*F* = 5.57, *p* = 0, η^2^ = 0.11). Comparisons between the SGS and ISS groups revealed significant differences when compared to the IPI group.

**Table 6:** Descriptive statistics, group differences, and effect sizes for the Physical Activity Enjoyment Scale (18–126 points) of each protocol.

				Mean difference (95% CI)						
	*M*	SD	Δ%	MD	LBCI	UBCI	*P*-value	F value	*P*-value	η^2^
IPI	106.10	17.40						5.57	0	0.11
SGS	116.10	10.20	9.4%	−9.36	−16.2	−2.55	0			
ISS	115.10	10.90	8.5%	−10.4	−17.2	−3.55	0			

IPI, Group with prescribed intensity (64–76% HRmax) (Liguori et al., 2021); SGS, Small Group with self-selected intensity; ISS, Individual Group with self-selected intensity. M, mean; SD, standard deviation; Δ%, % difference in M compared to the control group; F, F-ratio value; p, probability of F; η^2^, eta-squared effect size; CI, confidence interval; LBCI, lower bound of the 95% confidence interval; UBCI, upper bound of the 95% confidence interval; MD, mean difference.

**Figure 6:**
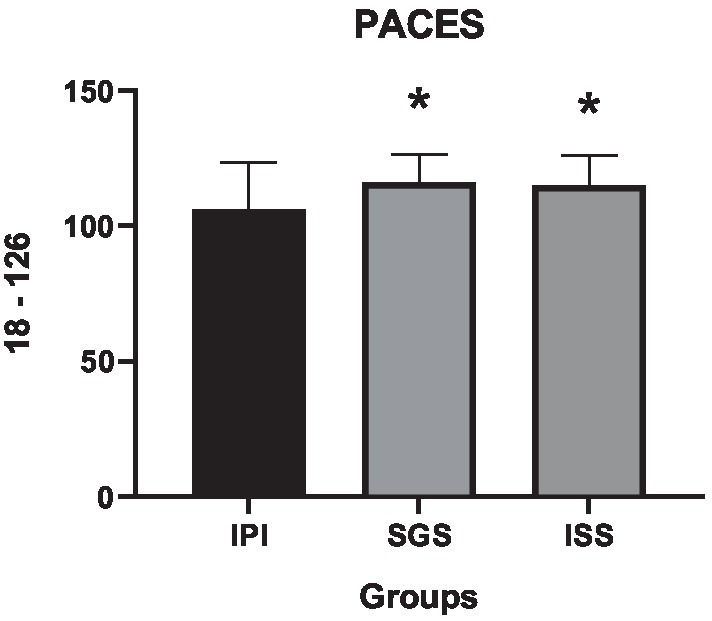
Means and standard deviations of the Physical Activity Enjoyment Scale (PACES) (vertical axis) compared between the control and experimental groups (horizontal axis). * = difference *p* < 0.05 compared to the IPI group.

The SGS group exhibited a 9.4% increase in the mean PACES score administered at the end of the exercise session (*p* = 0, mean difference = −9.36, 95% CI = −16.17 to −2.55), while the ISS group showed an 8.5% higher mean compared to the IPI group (p = 0, mean difference = −10.36, 95% CI = −17.17 to −3.55). In support of our secondary hypothesis, the PACES results were higher in both the SGS and ISS groups, indicating that self-selected intensity is more enjoyable in both individual and small group settings for women with overweight and obesity starting aerobic training programs, as illustrated in Figure 6.

The analysis of the Intention to Repeat the Exercise Session (INT) is presented in Table 7. The one-way ANOVA revealed a moderate interaction effect among the different groups (*F* = 18.6, *p* = 0, η^2^ = 0.39).

**Table 7:** Descriptive statistics, group differences, and effect sizes for the intention to repeat the exercise session (1–7 points).

				Mean difference (95% CI)						
	*M*	SD	Δ%	MD	LBCI	UBCI	*p*-value	*F* value	*p*-value	η^2^
IPI	4	1						18.6	0	0.39
SGS	6	1	41.6%	−1.8	−2.32	−1.27	0			
ISS	6	1	37.6%	−1.63	−2.25	−1.1	0			

IPI, Group with prescribed intensity (64–76% HRmax) (Liguori et al., 2021); SGS, Small Group with self-selected intensity; ISS, Individual Group with self-selected intensity. M, mean; SD, standard deviation; Δ%, % difference in M compared to the control group; F, F-ratio value; *p*-value, probability; η^2^, eta-squared effect size; CI, confidence interval; LBCI, lower bound of the 95% confidence interval; UBCI, upper bound of the 95% confidence interval; MD, mean difference.

**Figure 7:**
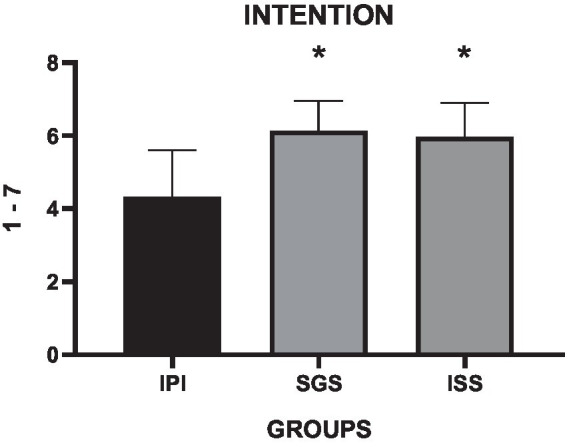
Means and standard deviations of the Intention to Repeat the Exercise Session scale (vertical axis) compared between the control and experimental groups (horizontal axis). * = difference *p* < 0.05 compared to the IPI group.

The mean values also revealed significant differences when compared to the control group. The SGS group exhibited a 41.6% increase in the mean INT score administered at the end of the exercise session (*p* = 0, mean difference = 1.8, 95% CI = −2.32 to −1.27), while the ISS group demonstrated a 37.6% higher mean INT compared to the control group (p = 0, mean difference = −1.63, 95% CI = −2.25 to −1.1). In support of our secondary hypothesis, the INT results were higher in both the SGS and ISS groups compared to the control group. These findings suggest that self-selected intensity increases the intention to repeat the exercise session three times in the following week, both in individual and small group conditions. This approach represents a training strategy that enhances the intention to repeat the behavior in the future for women with overweight or obesity who are initiating physical training programs, as depicted in Figure 7.

The analyses of the BPNSFS are presented in Table 8. The one-way ANOVA showed a large interaction effect among the different groups for autonomy (*F* = 290, p = 0, η^2^ = 0.87).

**Table 8:** Descriptive statistics, group differences, and effect sizes for basic psychological needs (6–30 points).

	IPI		SGS			ISS					
	*M*	SD	*M*	SD	Δ%	*M*	SD	Δ%	*F* value	*p*-value	η^2^
Autonomy	4.76	1.07	16.36	2.72	243.70%	16.83	2.43	253.60%	290	0	0.87
Relationship	5.30	1.14	16.26	2.37	206.80%	6.00	1.68	13.20%	346	0	0.88
Competence	15.56	3.19	14.36	3.17	−7.70%	15.08	3.36	−3.10%	1,07	0,34	0.02

Mean difference (95% CI)			Mean difference (95% CI)	
MD	LBCI	UBCI	MD	LBCI
−11.60	−12.90	−10.20	12.00	10.70
−10.96	−12.07	−9.85	−0.70	−1.81
1.20	−0.86	3.26	0.23	−1.83

IPI, Group with prescribed intensity (64–76% HRmax) (Liguori et al., 2021); SGS, Small Group with self-selected intensity; ISS, Individual Group with self-selected intensity. M, mean; SD, standard deviation; Δ%, % difference in M compared to the control group, *F* value, F-ratio value; *p*-value, probability of *F*; η^2^, eta-squared effect size; CI, confidence interval; LBCI, lower bound of the 95% confidence interval; UBCI, upper bound of the 95% confidence interval; MD, mean difference.

**Figure 8:**
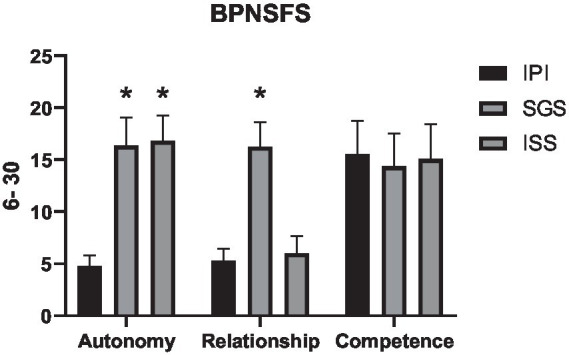
Means and standard deviations of the Basic Psychological Needs Satisfaction Scale (vertical axis) compared between the control and experimental groups (horizontal axis). * = difference *p* < 0.05 compared to the IPI group.

The mean values also revealed significant differences when compared to the control group. The SGS group demonstrated a 16.36% increase in mean autonomy at the end of the exercise session (*p* = 0, mean difference = 1.8, 95% CI = −2.32 to −1.27), while the ISS group exhibited a 16.83% higher mean compared to the control group (p = 0, mean difference = −1.63, 95% CI = −2.25 to −1.1). In support of our secondary hypothesis, the autonomy scores were higher in both the SGS and ISS groups compared to the control group. These findings suggest that self-selected intensity enhances the perception of autonomy in both individual and small group settings when compared to the control group, as shown in Figure 8.

The one-way ANOVA revealed a large interaction effect between the groups for relatedness (*F* = 346, *p* = 0, η^2^ = 0.88). The SGS group demonstrated a 206.8% increase in mean relatedness at the end of the exercise session (*p* = 0, mean difference = −10.96, 95% CI = −12.07 to −9.85), while the ISS group exhibited a 13.2% higher mean compared to the control group (*p* = 0.29, mean difference = −0.7, 95% CI = −1.81 to 0.29). This suggests that the ISS group had similar relatedness perceptions to the IPI.

The one-way ANOVA also revealed a small interaction effect for competence (*F* = 1.07, *p* = 0.34, η^2^ = 0.02), with means showing minimal differences across the protocols. The SGS group demonstrated a 7.7% lower mean competence compared to the control group at the end of the exercise session (*p* = 0.35, mean difference = 1.20, 95% CI = 0.86 to 3.26), while the ISS group had a 3.1% lower mean competence compared to the control group at the end of the session (*p* = 0.96, mean difference = 0.23, 95% CI = −1.83 to 2.3). These results suggest that all groups had similar competence perceptions by the end of the exercise session.

Thus, the results indicate that self-selected intensity in both groups (SGS and ISS) positively impacted autonomy perception compared to the control group. The SGS group demonstrated more favorable results for relatedness perception compared to both the IPI and the ISS groups.

The SGS protocol elicited a higher response in the relatedness variable, which may be attributed to the perception of social bonding and support experienced in a small group exercise context. Such an environment facilitates positive social interactions and fosters a sense of belonging among participants, aligning with the relatedness need as defined by Self-Determination Theory (Deci and Ryan, 2000). This theory posits that social connection constitutes a fundamental pillar of intrinsic motivation, thereby enhancing engagement and promoting long-term adherence to exercise programs. These findings underscore the significance of integrating social components into exercise prescriptions, particularly for populations that may derive substantial benefits from increased social support and a strengthened sense of community.

For competence perception, no significant differences were observed, with all groups showing similar values.

## 4 Discussion

The present study posits as its primary hypothesis that SGS and ISS would select an intensity similar to the control group (IPI), which followed the American College of Sports Medicine (Liguori et al., 2021) recommendations for moderate-intensity aerobic exercise prescription (64–76% of HRmax). The results support our primary hypothesis, as the mean heart rate values were similar across groups: control group IPI = 70.5% of HRmax, SGS group = 70.3% of HRmax, and ISS group = 71% of HRmax (*p* = 0.31, η^2^ = 0.02). Our findings align with previous studies, such as Lind et al. (2005), which explored the relationship between self-selected intensity and prescribed moderate intensities. The results indicated that when allowed to choose, participants generally selected intensities similar to those recommended, suggesting that self-selection can align with prescribed intensity in terms of physiological benefits.

We did not find studies in the literature comparing total distance covered (training volume) in aerobic exercise between prescribed and self-selected intensities. Our study addresses this gap by showing that the volume load covered was similar across groups (*p* = 0.45, *F* = 0.87, η^2^ = −0.03), with the SGS group covering 3.8% less distance than IPI (*p* = 0.28, mean difference = 0.08, 95% CI = −0.72 to 0.23). On the other hand, the ISS group covered 2.9% less distance than IPI (*p* = 0.12, mean difference = −0.35, 95% CI = −0.35 to 0.27).

In this context, we consider that self-selected intensity in aerobic exercise is a suitable prescription approach both from a physiological standpoint for sedentary adult women with overweight/obesity and in terms of affective responses (Ekkekakis and Lind, 2005; Parfitt et al., 2006; Lind et al., 2008; Ekkekakis et al., 2005). The evaluation of middle-aged sedentary women by Lind et al. (2005) showed that self-selected intensity corresponded to the prescribed recommendation for improving aerobic capacity, indicating that self-selection can achieve levels similar to those prescribed for this population.

In line with the tripartite exercise model of Ladwig et al. (2017), the dimensions of efficacy, safety, and pleasure support the hypotheses of the present study. Traditionally, exercise prescriptions focus on efficacy, ensuring that exercise promotes desired physiological adaptations, and safety, minimizing the risk of injury. However, low adherence to gym exercise programs is a widely recognized challenge (Sperandei et al., 2016; Gjestvang et al., 2019; Gjestvang et al., 2020; Gjestvang et al., 2021; Gjestvang et al., 2023), with dropout rates within the first 3–6 months, suggesting that current strategies may hinder adherence.

For older adults with chronic conditions, factors such as physical limitations and underlying health issues can pose significant barriers to participation in physical activities. Nevertheless, structured aerobic programs, such as walking, have been shown to enhance cognitive functioning and quality of life, emphasizing the potential of tailored interventions to improve exercise enjoyment and adherence (Cohen, 1988). These findings are consistent with our study, which demonstrated that small-group exercise elicited greater enjoyment compared to the IPI protocol. This highlights the critical role of social interaction in promoting positive affective responses and supporting sustained engagement in physical activity programs.

The perception of pleasure during exercise is a factor that optimizes long-term adherence, as enjoyable experiences during exercise generate positive affective responses associated with maintaining future behavior and intention to repeat exercise (Williams et al., 2016; Williams, 2008; Rhodes and Kates, 2015; Williams et al., 2008).

Our secondary hypothesis posits that self-selected intensity protocols (SGS and ISS) would result in higher affective responses compared to IPI. Our data showed that affective valence was higher in SGS and ISS (*p* = 0, *F* = 5.72, η^2^ = 0.09), with SGS showing 73.1% higher exercise enjoyment compared to IPI (*p* = 0, mean difference = 0.78, 95% CI = −1.1 to −0.45). In ISS, affective valence during exercise was 98.8% higher than IPI (p = 0, mean difference = −1.02, 95% CI = −1.34 to 0.7).

Our findings are consistent with Ekkekakis and Lind (2005), who evaluated overweight and obese adults performing aerobic exercise sessions and found that affective valence in self-selected intensity was more positive than in prescribed intensity. Thus, we suggest that self-selection of aerobic exercise intensity should consider not only efficacy and safety but also the pleasurable nature of training. Parfitt et al. (2006) investigated sedentary adults in self-selected and prescribed intensity aerobic training with heart rate and pleasure/displeasure monitoring, demonstrating that self-selected intensity resulted in higher affective responses than prescribed intensity.

The Feeling Scale is a reliable tool for assessing self-selected intensity in aerobic training, especially for women in gym environments. Hamlyn-Williams et al. (2015) demonstrated that sedentary women effectively used the FS to regulate exercise intensity at moderate levels, predominantly experiencing positive affective responses. These findings suggest that the FS allows for real-time effort adjustment according to one’s emotional and physical state, promoting a more enjoyable experience. The potential of using the FS as an accessible tool for regulating exercise intensity appears to support and contribute to a pleasant exercise practice tailored to individual preferences.

As a mediator of successful interventions aimed at exercise continuity, enjoyment is associated with pleasurable experiences that increase the likelihood of adherence to training (Jekauc, 2015). Positive and enjoyable exercise experiences increase the probability of maintaining active behavior in the future, with previously experienced pleasure being a strong predictor of adherence (Rodrigues et al., 2021). The results of the present study showed that enjoyment was higher in the self-selected intensity groups SGS and ISS (*p* = 0, *F* = 5.57, η^2^ = 0.11), with SGS 9.4% higher than IPI (p = 0, mean difference = 9.36, 95% CI = −16.2 to −2.55) and ISS 8.5% higher than IPI (p = 0, mean difference = 10.4, 95% CI = −17.2 to −3.55).

In this context, we understand that self-selected intensity results in greater enjoyment in adult women with overweight/obesity, indicating that it is a positive strategy for optimizing affective responses during exercise, as enjoyment is a predictor of future behavior related to physical exercise. Rodrigues et al. (2021) investigated how enjoyment influences exercise adherence, associating enjoyment with previous experiences and current exercise adherence. The results showed that enjoyment experienced in previous exercise sessions was a strong predictor of continuous adherence.

The experience of pleasure or discomfort during activity influences future intention for continued or discontinued exercise practice. The hedonic theory of human behavior associates that individual who maximize pleasure and minimize discomfort during exercise (Kahneman, 1999) have greater long-term adherence to training. Perceived pleasure fosters greater intrinsic motivation (Deci and Ryan, 2000), increasing the likelihood of adherence to sustained training commitment. The present study showed that the intention to engage in physical exercise three times in the following week, an indicator of future behavior, was higher in the SGS and ISS protocols compared to IPI (*p* = 0, *F* = 18.6, η^2^ = 0.39). In SGS, the intention to repeat the exercise session three times in the following week was 41.6% higher (*p* = 0, mean difference = 1.8, 95% CI = −2.32 to −1.27) than IPI, while ISS was 37.6% higher than IPI (*p* = 0, mean difference = 1.63, 95% CI = −2.25 to −1.1). The results of the present study, based on the hedonic theory of human behavior (Ekkekakis, 2003), demonstrate that self-selected protocols were more positive in affective valence and enjoyment, suggesting better results in the intention to repeat the exercise session the following week (future behavior).

Our study suggests that self-selected exercise intensity may yield positive psychological and physiological responses, optimizing affective responses such as pleasure and reducing discomfort when compared to rigid exercise prescriptions. These findings align with those of Ekkekakis (2003), who demonstrated that more pleasant exercise experiences support long-term adherence, as well as promoting positive emotional experiences during training (Ekkekakis, 2009; Ekkekakis and Lind, 2005; Ekkekakis, 2003; Ekkekakis et al., 2006).

Given that adherence and continuity in exercise programs are influenced by motivational factors, Self-Determination Theory (Deci and Ryan, 2000) posits that human motivation and well-being are largely shaped by the satisfaction of basic psychological needs: autonomy, competence, and relatedness. Our results indicated that the perception of autonomy was significantly higher in the SGS and ISS groups compared to the IPI group (*p* = 0, *F* = 290, η^2^ = 0.9). Specifically, the SGS group exhibited a 243.7% increase (*p* = 0, mean difference = −11.6, 95% CI –12.9 to −10.2), and the ISS group showed a 253.6% increase (p = 0, mean difference = 12, 95% CI 10.7 to 13.4) compared to IPI. Autonomy refers to the desire to feel responsible for one’s actions and choices, suggesting that allowing participants to self-select exercise intensity may be an effective strategy for fostering autonomy in exercise programs.

Regarding competence, which involves a sense of efficacy and skill development (Deci and Ryan, 2000), our results revealed no significant differences between groups (*p* = 0.34, *F* = 1.07, η^2^ = 0). The SGS group showed a 7.7% lower perception of competence compared to IPI, and the ISS group was 3.1% lower than IPI. Although competence is associated with intrinsic motivation, Self-Determination Theory (Deci and Ryan, 2000) highlights that the perception of efficacy is a stronger predictor of exercise behavior. These findings suggest that participants felt capable of successfully performing the exercise session, regardless of the method used to determine intensity.

When comparing small group and individual training, we anticipated changes in the relatedness component, which refers to the connection and sense of belonging to others (Deci and Ryan, 2000). Small group training promotes a positive social environment, creating a sense of support and community. Social interaction in small groups can optimize motivation and may contribute to the continuation of the exercise program (Wayment and McDonald, 2017). In our study, the relatedness values for SGS and ISS were 206.8 and 13.2% higher than IPI, respectively, with no significant differences observed. Autonomous motivation, which encompasses the satisfaction of the psychological needs for autonomy, competence, and relatedness, could explain the maintenance of future exercise behavior (Rodrigues et al., 2021; Teixeira et al., 2012; Deci and Ryan, 2000). Psychological needs strengthen intrinsic motivation, which is associated with personal satisfaction and well-being (Ekkekakis et al., 2011; Deci and Ryan, 2000; Rodrigues et al., 2021), and a motivational environment that fosters these needs supports sustained exercise engagement.

The results of this study support the initial hypotheses, showing that protocols focused on self-selecting exercise intensity are a practical and effective approach to promoting motivational experiences and positive affect in overweight and obese adult women. This positive effect appears to contribute to sustained exercise adherence, a critical factor in improving long-term health and quality of life. Furthermore, our findings demonstrate that self-selected intensities yield physiological effects comparable to traditional exercise prescriptions, with similar total training volumes across both self-selected and prescribed intensity conditions. To the best of our knowledge, this is the first study to analyze this aspect in the scientific literature.

The SGS and ISS protocols were designed to stimulate competence development through skill acquisition and progress, as well as enhance interpersonal relationships. We understand that social interactions within small groups provide a sense of belonging and social support—factors that should be integrated into exercise program prescriptions to increase enjoyment and the likelihood of long-term adherence (Teixeira et al., 2012; Deci and Ryan, 2000). The study’s results revealed mean differences of 1 to 2 units on the Feeling Scale between the SGS and ISS groups, confirming a positive affective response to self-selected intensity protocols. These findings suggest that positive experiences during physical exercise may increase future physical activity levels (Williams et al., 2008). A one-point increase on the FS corresponds to an additional 27–29 min of physical activity per week, with further increases of 15–38 min per week after 6 months (Williams et al., 2012), potentially reaching up to 41 additional minutes per week after 12 months (Williams et al., 2008).

This study fills a gap in the literature by demonstrating that, even within small group contexts, self-selected intensity results in training volumes similar to those prescribed in traditional exercise prescriptions (Liguori et al., 2021). In addition to preserving exercise efficacy and safety, the small group format enhances social relationships, a psychological construct associated with autonomy and crucial in regulating active behavior. The social aspect of small group training fosters a perception of support, which contributes to long-term adherence and engagement (Teixeira et al., 2012; Deci and Ryan, 2000).

Thus, the results of the present study suggest that affective indicators should be considered in the selection of exercise intensity and volume in aerobic training prescription guidelines. While these guidelines are widely recognized (Liguori et al., 2021) and support individualized exercise, their integration with psychological variables remains limited. The various factors analyzed in the SGS and ISS protocols provide new insights into exercise psychophysiology and present a practical, evidence-based approach to intensity prescription, consistent with ACSM (2021) recommendations (Liguori et al., 2021). This approach emphasizes the prioritization of emotional experience in practitioners (Wienke and Jekauc, 2016).

The SGS and ISS protocols employed in this study, which allowed participants to self-select their exercise intensity during aerobic exercise, resulted in higher perceived autonomy compared to the IPI group (*p* < 0.05). This indicates greater efficacy in the affective experience of exercise, factors which may promote health and improve physical fitness (Hamlyn-Williams et al., 2015; Costa et al., 2015). Among the strengths of the study are: (Shilton et al., 2024) the similarity in intensity and volume between the SGS and ISS groups, which suggests that small group training is a safe and enjoyable alternative to individual exercise at self-selected intensity, comparable to traditional prescription; and the importance of fostering autonomy and social relationships in the small group protocol, particularly when participants share similar demographics, such as age and sex, as in the present study.

Achieving sustainable behavior change requires longitudinal analyses, as participants may drop out or experience relapse over time. We believe that this study could inform future interventions that extend the monitoring period and track engagement with training sessions. Future research should investigate the efficacy of this intervention in longitudinal contexts, assessing adherence, frequency, and effects on fitness changes in supervised environments.

The main findings of the present study indicate that the intensity and training volume were comparable across the IPI, SGS, and ISS protocols, demonstrating that self-selected intensity is biologically equivalent to prescribed intensity. Affective responses, including pleasure, enjoyment, and intention to repeat the activity, were more positive in the self-selected intensity protocols. This underscores self-selection as a safe, effective, and more enjoyable alternative to prescribed intensity in aerobic training. Moreover, self-selection facilitated greater autonomy, a core construct for sustaining exercise behavior, as outlined by Self-Determination Theory. Small-group training with self-selected intensity emerged as an effective approach, enhancing both autonomy and relatedness—key components of Self-Determination Theory—which may contribute to improved adherence to aerobic exercise routines. These findings present practical strategies that can be implemented in fitness centers and public health programs to promote sustained physical activity participation.

The findings of the present study suggest that exercise professionals working with obese women should implement individualized, participant-centered strategies. Allowing participants to self-select exercise intensity within safe parameters can enhance feelings of pleasure and autonomy, while incorporating small-group sessions can strengthen social support and foster a sense of connection. Regular monitoring of affective responses, such as valence and pleasure, is crucial for adjusting sessions to optimize enjoyment and effectiveness. Furthermore, creating a supportive and inclusive environment, setting realistic and attainable goals, and ensuring gradual progression in exercise programs can enhance intrinsic motivation and promote long-term adherence, consistent with the principles of Self-Determination Theory.

A key limitation of the present study is its cross-sectional design and the small sample size, comprising overweight/obese adult women, which restricts the generalizability of the findings to other populations, such as men or younger individuals. Additionally, the short-term nature of the study and reliance on self-reported psychological measures represent notable limitations. While the findings suggest that self-selected intensity and small-group training promote positive affective experiences and may enhance exercise adherence, it remains uncertain whether these effects are generalizable to individuals with chronic conditions or to different age groups. Future research should investigate the efficacy of this psychophysiological approach in diverse demographic contexts. Furthermore, longitudinal studies are needed to assess the effectiveness of self-selected intensity in sustaining exercise behavior over time and across various exercise modalities, such as resistance training, to expand and solidify the practical applications of this strategy in promoting physical activity adherence.

## 5 Conclusion

The findings of the present study suggest that self-selected intensity in aerobic exercise, whether in individual or small group settings, is a feasible and more effective strategy for promoting positive affective experiences in overweight/obese women, without compromising standard recommendations for exercise intensity and volume. Self-selection not only results in intensity levels comparable to traditional prescriptions but also significantly increases perceived pleasure and the intention to repeat the exercise, factors that may enhance adherence to training.

Moreover, the small group model was equally effective, adding the benefit of fostering social relationships, which are linked to better behavioral regulation and more intrinsic motivation for exercise. These findings indicate that exercise prescription strategies incorporating autonomy and social support can be more effective, particularly for populations facing barriers to engagement in exercise programs, such as overweight/obese women.

In summary, this study advances our understanding of psychophysiological responses to aerobic exercise and underscores the importance of integrating pleasure and social support into exercise guidelines. This is especially relevant for groups that may benefit from a more supportive and flexible training environment tailored to individual preferences.

We thank CAPES, Coordination for the Improvement of Higher Education Personnel for promoting the research project and São Judas Tadeu University for providing the laboratory for data collection.

## Data Availability

The original contributions presented in the study are included in the article/Supplementary material, further inquiries can be directed to the corresponding author.
